# Rehabilitation With Eccentric Training Using Kettlebell and Kinesio Taping in a Young Volleyball Player With Proximal Biceps Tendinopathy: A Case Report

**DOI:** 10.7759/cureus.62887

**Published:** 2024-06-22

**Authors:** Pratik R Jaiswal, Swapnil U Ramteke, Priya Tikhile

**Affiliations:** 1 Sports Physiotherapy, Ravi Nair Physiotherapy College, Datta Meghe Institute of Higher Education & Research, Wardha, IND; 2 Musculoskeletal Physiotherapy, Ravi Nair Physiotherapy College, Datta Meghe Institute of Higher Education & Research, Wardha, IND

**Keywords:** young athlete, volleyball, training, kinesio taping, kettlebell, eccentric exercises, tendinopathy, proximal biceps

## Abstract

A prevalent overuse injury among athletes, especially to those participating in sports like volleyball that demand repeated overhead motions, is biceps tendinopathy. The painful condition known as biceps brachii tendinopathy is characterized by changes in the structure of the tendon together with chronic degeneration. Furthermore, the biceps aid in the acceleration and deceleration of the arm in numerous overhead sports. The biceps may experience excessive strain as a result of poor training or exhaustion. It is commonly known that the long head of the biceps tendon plays a significant role in producing pain, particularly when it comes to anterior shoulder discomfort and dysfunction in athletes and working people. Athletes' biceps tendon conditions fall into three broad categories: degeneration, instability, and abnormalities that are of source. This case details the use of kettlebell eccentric exercise and kinesio taping in the rehabilitation of a young volleyball player with biceps tendinopathy. The four primary aspects of the rehabilitation regimen were kinesio taping, pain management and rest, eccentric training with kettlebells, and initial assessment and patient education. Kettlebell eccentric training was used to enhance eccentric strength and encourage tendon remodeling, and kinesio taping was used to give pain relief and structural stability. This case study emphasizes the value of an all-encompassing rehabilitation strategy catered to the unique requirements of every athlete by demonstrating the effectiveness of kinesio taping and eccentric exercise with kettlebells in the treatment of biceps tendinopathy.

## Introduction

In sports like handball, volleyball, and swimming, where motions are routinely carried out above the head at high speeds or in awkward angles, shoulder injuries are common. These athletes are known as "overhead athletes." It is commonly acknowledged that they have a high chance of suffering from shoulder discomfort, and numerous studies have been done to determine contributing variables and provide the best possible care [1]. The swiftest athletic activity used in sports is the overhead throwing action [2]. Tendinopathy is a long-term condition where there is an immunological reaction imbalance affecting tendons. It is characterized by signs of degeneration and little involvement of inflammation, resulting in failure to cure the tendons. As a result, it has no single perfect way to cure tendinopathy. When the shoulder moves, the tendinous tissue in the long head of the biceps (LHB) becomes micro-traumatized due to repeated, chronic tension and friction stresses [3]. There is ongoing discussion over the functioning of the LHB and most significantly, how best to treat its problems with a great deal of studies looking into the internal structure of the LHB and the several clinical illnesses that impact it [4]. Although there are nonoperative alternatives for less severe lesions or mild symptoms like tendinopathy or partial LHB tears, surgery is a suitable treatment for lesions of the biceps, superior labrum anterior-posterior, LHB partial tears, and LHB subluxation. Though biceps tenotomy and tenodesis are the two preferred techniques, the best medical treatment for LHB tendon injuries is still up for debate [5]. The biomechanics requirements and high tension in which overhead athletes impose on their shoulders can lead to pathological alterations in the biceps tendons and even ruptures. The evaluation of the root cause of pathological alterations in the biceps might be difficult due to the strong relationship between biceps lesions and associated shoulder issues [6]. The tendon runs via the rotator interval, the coracohumeral ligament, and the bicipital groove as it exits the glenohumeral joint. Here, the tendon passes underneath the falciform process, the aponeurotic extension of the pectoralis major, and the transverse humeral ligament, which spans the space between the greater and lesser tuberosities. The mean depth of the groove is minimal over 4 mm, and the mean medial wall angle is 56° [7].

As the tendon is divided into the intra-articular and extra-articular sections, the anatomy of the LHB is distinct. The intra-articular portion, which runs beneath the rotator cuff and attaches to the superior labrum and glenoid, is further vulnerable to trauma, particularly when it comes to rupturing of adjacent tissues. Examples of this include rotator cuff tears, superior labrum anterior to posterior tears, and subacromial bursa inflammation that results in subacromial impingement [8]. For a prolonged time, intra-articular LHB tendon lesions have been recognized as important sources of shoulder pain [9]. Tendinitis is the term used to describe inflammation within the biceps tendon. But since there isn't actually any inflammation involved in the illness, it's more fair to refer to it as tendinosis or tendon degeneration. Collagen fiber wasting, asymmetric collagen fiber patterning with tendon fissuring, fibrinoid necrosis, and fibrocyte proliferation are some of the micro alterations. The primary source of biceps deterioration is believed to be the friction of the tendon with the coracoacromial arch due to the close relationship between the biceps tendon and the rotator cuff [10]. When an athlete engages in overhead activity, their shoulder is frequently under the strain. It is possible to gain greater knowledge of biceps disorders and biceps functioning in shoulders having pathologic modifications by comprehending the variations of muscular activity of the biceps during the overhand pitch. According to an electromyographic examination of the biceps during a throw, the biceps in a normal shoulder primarily works as an elbow flexor while pitching, acting in a manner similar to the brachialis [11].

At follow-through, while controlled arm deceleration is necessary to prevent a hyperextension snap at the elbow, peak biceps activity is observed [12]. The stresses produced by repeated overhead exercise may eventually exceed the shoulder's anterior static constraints' capacity to adjust leading to an unstable impingement complex that is associated with glenohumeral instability. Progressive relaxation of these constraints may ultimately result in a traction damage to the biceps tendon and rotator cuff, which will wear you out physically. While it happens, provocative arm postures may cause a small rise in anterior and superior translation of the humeral head, which could result in secondary impingement of the rotator cuff and possibly the biceps [13]. The benefits of eccentric training for the rehabilitation of tendinopathies of the Achilles, patellar, and, relatively recently, upper limb tendinopathies, notably those affecting the rotator cuff and wrist extensor process, have been well-documented. Patients suffering from such tendinopathies may see improvements in their tendon properties, activities, and discomfort through eccentric training [14].

## Case presentation

Patient information

A 17-year-old male volleyball player visited the physiotherapy division for concerns regarding anterior shoulder pain and sometimes at the biceps belly of the right side. He reported experiencing the pain for the past 20 days, with manifestation being minor before playing and deteriorating during and after playing. The athlete has been playing for the last six years. The pain had a gradual onset and progressively worsened, particularly intensified by overhead smashing and overhead daily tasks. Notwithstanding pain medications, the signs continued, leading to referral to the physiotherapy department. The patient's height was 166 cm, and he weighed 58 Kg. He had been taking analgesics (ibuprofen) to counter pain but got no relief. He reported no significant previous pathologies or history of surgeries. Additionally, no other medical conditions were reported. His family medical history was nonsignificant for similar conditions. The patient's lifestyle includes a regular training regimen consistent with competitive volleyball players, involving both on-court and strength training sessions.

Clinical assessment

Tenderness was present as direct palpation over the bicipital groove elicited a painful response. Upon special test examination, speed test was positive as the painful response was elicited in the bicipital groove when the patient attempted to forward flex the shoulder against the examiner’s resistance with the elbow slightly flexed and the forearm supinated. In the Yergason test, the arm was stabilized against the patient’s trunk, and the elbow was flexed to 90° with the forearm pronated. Upon manual resistance to supination, the patient also externally rotated the arm against resistance. Painful response was reported over the bicipital groove without any pop on palpation. The patient also underwent the Hawkins-Kennedy test, which was positive, indicating subacromial impingement. Additionally, the O'Brien test was performed to rule out a superior labrum anterior-posterior lesion, and it was negative, thereby reducing the likelihood of this associated condition.

The individual described an insistent dull ache, rating it as 5/10 at rest and 8/10 during activity on the numerical pain rating scale. Range-of-motion evaluation of the right shoulder is in Table 1 and manual muscle testing is in Table 2. Table 3 shows outcome measures.

**Table 1:** Range-of-motion assessment of the right shoulder joint

Movement	Pre-intervention		Post-intervention	
	Active	Passive	Active	Passive
Flexion	0-110	0-115	0-170	0-175
Extension	0-40	0-45	0-55	0-60
Abduction	0-120	0-125	0-160	0-165
Adduction	0-30	0-35	0-40	0-45
Internal rotation	0-45	0-50	0-65	0-70
External rotation	0-50	0-55	0-70	0-75

**Table 2:** Manual muscle testing of the right shoulder

Muscles	Pre-intervention	Post-intervention
Shoulder flexors	3/5	4/5
Shoulder extensors	3/5	4/5
Shoulder abductors	3/5	4/5
Shoulder adductors	3/5	4/5
Shoulder internal rotators	3/5	4/5
Shoulder external rotators	3/5	4/5

**Table 3:** Outcome measures

Outcome measure	Pre-intervention	Post-intervention
Visual analogue scale	8/10	0/10
Quick dash (work module)	18.75%	0%
Quick dash (sports module)	68.75%	0%

Therapeutic intervention

**Figure 1:**
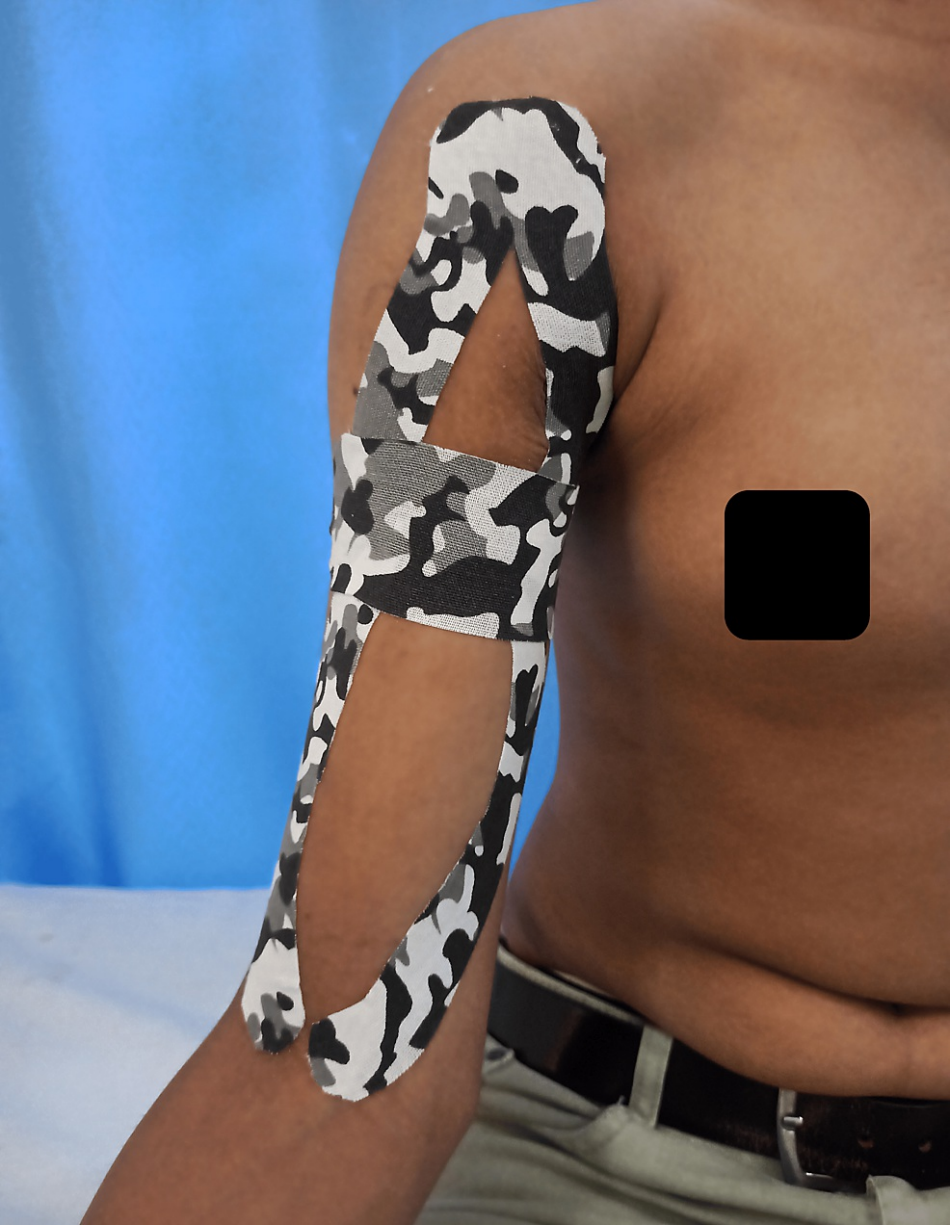
Kinesio taping for bisceps tendinopathy

The phasic intervention is shown in Tables 4-6. Figure 1 shows kinesio taping.

**Table 4:** Intervention for week 1 NA: not applicable; LIPUS: low-intensity pulsed ultrasound; MHz: megahertz; W: watt

Goal	Intervention	Rationale	Dosage
Patient education	Correct posture was emphasized during the patient's rehabilitation to optimize shoulder mechanics and reduce strain on the biceps tendon. Specific instructions included maintaining a neutral spine position, which involves aligning the ears, shoulders, and hips vertically while avoiding excessive curvature of the spine. The patient was taught to avoid protracting the scapula. Also, the patient was instructed to retract and depress the scapulae, gently pulling the shoulder blades back and down toward the spine. This posture helps in reducing undue stress on the shoulder joint and biceps tendon, thereby aiding in the recovery process	To provide proximal stability to enable appropriate movement distally	NA
	Avoidance of activities that exacerbate symptoms	Essential for effective pain management, prevention of further damage, and facilitation of healing without undue stress	NA
Pain management	LIPUS	Nonthermal and/or nondestructive thermal actions to quicken the recovery process. Boosting collagen alignment, improving tissue biomechanical characteristics, and promoting growth of cells	1 MHz, 1.5 W/cm2, continuous mode, 4 min daily
	Kinesio taping	For structural support, alleviate pain and facilitate proprioception	New tape was applied every alternate day

**Table 5:** Rehabilitation for weeks 2-4 kg: kilogram

Goal	Intervention	Rationale	Dosage
Eccentric strengthening	Isometric holds with shoulder at multiple level of flexion and elbow in slight flexion with 2 kg kettlebell	To effectively stimulate tendon adaptation and promote collagen synthesis, essential for tendon healing and remodeling	2-3 times a week, three sets of 10 to 15 reps initially. Gradually increasing intensity and volume as tolerated with proper technique and avoiding compensatory movements
	Eccentric biceps curls with elbow in supination focusing on slow lowering phase (3-5 seconds) with lighter weights initially		
	Eccentric loading of biceps with elbow in mid-prone focusing on slow lowering phase (3-5 seconds) with lighter weights initially		

**Table 6:** Rehabilitation for weeks 5-6

Goal	Intervention	Rationale
Functional rehabilitation	Progressive resistance training by kettlebell swings, accelerated swings & goblet squats	Incorporating sport-specific exercises and drills ensures that the athlete is adequately prepared for the demands of volleyball, reducing the likelihood of recurrence and promoting long-term athletic success. Routine observation as well as progression of the rehabilitation program is necessary to maximize results as well as promote a safe return to sport
	Volleyball-specific movement drills such as hitting, blocking, and serving, performed at submaximal intensity to reinforce proper technique and neuromuscular control	

## Discussion

A major treatment concern for athletes is biceps tendinopathy, especially for individuals who play sports like volleyball that require frequent overhead actions. In this rehabilitation protocol, the use of kettlebells and eccentric training is in line with the most recent research that supports the use of eccentric exercise as a mainstay in treating tendinopathies [15]. In accordance with earlier research that suggested kinesio tape may be useful in treating tendinopathies, kinesio taping was used to relieve pain and give the injured tendon extra assistance. The biceps tendon and surrounding musculature were covered with kinesio tape to relieve tension, lessen strain during movement, and increase proprioception. All of which can lead to pain reduction and improved performance [16].

Through a cadaveric investigation, Eames et al. confirmed how the tendon of the LHB, implanted on the tuberosity distant from the forearm's rotational axis, is a superior supinator, while the short head, attached distal to the radial tuberosity, was a higher potent elbow flexor. The forearm's posture influences these biceps functions. It's common knowledge that while the forearm is prone, the biceps muscle exhibits decreased activity. As the forearm is supinated, the flexor role can increase, and the biceps take on the role of the main forearm supinator as the elbow gradually flexes to a 90° angle [17]. A rehabilitation regimen for biceps tendinopathy was developed by Krupp et al. and consists of pain management, full passive range-of-motion restoration, active range of motion, and strength training [18]. Common treatments include rest with an arm sling, injections of corticosteroid and nonsteroidal anti-inflammatory drugs, and manual or ultrasound-guided physical therapy (stretching, strengthening of the scapular muscles, restoring of the glenohumeral joint's complete range of motion, and enhancement of movement pattern) [19].

When referring to overuse tendon injuries in athletes, majority of authors have favored using the term tendinopathy rather than tendinitis. The developing indication of histological classification, which is characterized by tendon degradation without symptoms of inflammation, takes precedence over these guidelines regarding language. Having a deeper understanding of the variations in tendon pathology can help clinicians treat these disorders more effectively [20]. Tenocyte metabolism is thought to be accelerated by the impact of tissues, which could hasten repair [21]. Because eccentric training restores the normal structure of the tendons, it has been demonstrated to be effective in treating tendinopathy. Eccentric exercise has been shown in multiple trials to provide beneficial results for upper quarter tendinopathies, namely, involving the rotator cuff and wrist extensor mechanism. Given the favorable results of treating other upper quarter tendinosis and the lack of evidence of harmful effects, physiotherapists may find it suitable to try eccentric training for patients with distal biceps tendinopathy as the fundamentals of tissue healing are similar [22].

## Conclusions

In conclusion, a young volleyball player with biceps tendinopathy showed encouraging results in pain relief, strength enhancement, and function restoration with the rehabilitation regimen that included eccentric training with kettlebells and kinesio taping. The athlete was able to recover safely with this regimen which included progressive loading, pain management, and functional rehabilitation. Kettlebell training combined with eccentric training has proven to be an efficient way to promote collagen production and tendon adaptability, both of which are necessary for tendon remodeling and healing. Furthermore, kinesio taping provided proprioceptive input, pain alleviation, and structural support, improving the ability to perform functional motions and minimizing the possibility of re-injury. Restoring sport-specific movement patterns, enhancing neuromuscular control, and getting the athlete ready for volleyball requirements were all made possible by functional therapy. In order to attain the best outcomes in the treatment of biceps tendinopathy, this case study emphasizes the significance of customized rehabilitation plans made to meet the particular requirements specific to each athlete. It also underlines the significance of a multidisciplinary approach.
